# Using Drosophila to Identify Naturally-Occurring Modifiers of Alzheimer's Disease

**DOI:** 10.1093/geroni/igab046.2425

**Published:** 2021-12-17

**Authors:** Adrienne Wang, Ming Yang, Cecilia Fitzgerald-Cook, Ben Harrison, Akimi Green, Kensington Hartman, Matthew Zinkgraf, Daniel Promislow

**Affiliations:** 1 Western Washington University, Bellingham, Washington, United States; 2 University of Washington, Seattle, Washington, United States

## Abstract

Despite significant progress in identifying risk factors for late-onset Alzheimer’s Disease (LOAD), much of the variance in disease pathogenesis remains unexplained, likely due to the contribution of many genes of small effect size. Model organisms such as Drosophila Melanogaster exhibit conservation in both disease-causing genes and cellular processes implicated in Alzheimer’s Disease (AD), offering a genetically tractable model that can be statistically leveraged to identify causal variants. Here, we combine a Drosophila model of AD with the Drosophila Genetic Reference Panel (DGRP), a model of natural variation consisting of over 200 fully sequenced, isogenic lines derived from a wild-caught population. Expression of two proteins closely associated with AD pathogenesis, A□42 and Tau, in the Drosophila eye results in a “rough eye” phenotype, an easily quantifiable phenotype caused by degeneration of the ommatidial array. By quantifying the degree of A□42- and Tau-mediated degeneration across 164 lines of the DGRP and using a gene-based approach to map associations, we have identified and validated a subset of naturally occurring modifiers of degeneration in Drosophila. Enrichment analysis reveals that the set of genes identified in our screen show significant enrichment for genes identified as significant or suggestive (4x10-6>p>2x10-11) in human GWAS studies. The results presented here provide proof-of-principal for an approach that combines the strengths of forward genetic screens in model organisms with the power of human GWAS studies to identify and validate potential risk factors that have been difficult to detect in human studies alone.

